# Measuring Physical Activity in a Cardiac Rehabilitation Population Using a Smartphone-Based Questionnaire

**DOI:** 10.2196/jmir.2419

**Published:** 2013-03-22

**Authors:** Leila Pfaeffli, Ralph Maddison, Yannan Jiang, Lance Dalleck, Marie Löf

**Affiliations:** ^1^The National Institute for Health InnovationUniversity of AucklandAucklandNew Zealand; ^2^Department of Sport and Exercise ScienceUniversity of AucklandAucklandNew Zealand; ^3^Department of Biosciences and NutritionKarolinska InstitutetStockholmSweden

**Keywords:** cellular phone, self report, motor activity, bias, cardiovascular diseases

## Abstract

**Background:**

Questionnaires are commonly used to assess physical activity in large population-based studies because of their low cost and convenience. Many self-report physical activity questionnaires have been shown to be valid and reliable measures, but they are subject to measurement errors and misreporting, often due to lengthy recall periods. Mobile phones offer a novel approach to measure self-reported physical activity on a daily basis and offer real-time data collection with the potential to enhance recall.

**Objective:**

The aims of this study were to determine the convergent validity of a mobile phone physical activity (MobilePAL) questionnaire against accelerometry in people with cardiovascular disease (CVD), and to compare how the MobilePAL questionnaire performed compared with the commonly used self-recall International Physical Activity Questionnaire (IPAQ).

**Methods:**

Thirty adults aged 49 to 85 years with CVD were recruited from a local exercise-based cardiac rehabilitation clinic in Auckland, New Zealand. All participants completed a demographics questionnaire and underwent a 6-minute walk test at the first visit. Subsequently, participants were temporarily provided a smartphone (with the MobilePAL questionnaire preloaded that asked 2 questions daily) and an accelerometer, which was to be worn for 7 days. After 1 week, a follow-up visit was completed during which the smartphone and accelerometer were returned, and participants completed the IPAQ.

**Results:**

Average daily physical activity level measured using the MobilePAL questionnaire showed moderate correlation (*r*=.45; *P*=.01) with daily activity counts per minute (Acc_CPM) and estimated metabolic equivalents (MET) (*r*=.45; *P*=.01) measured using the accelerometer. Both MobilePAL (beta=.42; *P*=.008) and age (beta=–.48, *P*=.002) were significantly associated with Acc_CPM (adjusted *R^2^*=.40). When IPAQ-derived energy expenditure, measured in MET-minutes per week (IPAQ_met), was considered in the predicted model, both IPAQ_met (beta=.51; *P*=.001) and age (beta=–.36; *P*=.016) made unique contributions (adjusted *R^2^*=.47, *F*_2,27_=13.58; *P*<.001).There was also a significant association between the MobilePAL and IPAQ measures (*r*=.49, beta=.51; *P*=.007).

**Conclusions:**

A mobile phone–delivered questionnaire is a relatively reliable and valid measure of physical activity in a CVD cohort. Reliability and validity measures in the present study are comparable to existing self-report measures. Given their ubiquitous use, mobile phones may be an effective method for physical activity surveillance data collection.

## Introduction

The case for a new technology to measure physical activity is compelling. Participation in regular physical activity is associated with a plethora of positive physical and mental health outcomes [1-3], and the burden associated with physical inactivity is considerable [4,5]. Much of the data supporting the commonly known benefits of physical activity are based on self-reported measures of physical activity. Physical activity questionnaires are commonly used to assess physical activity in large population-based studies because of their low cost and convenience [6]. Although many of the self-report questionnaires used in these studies have shown to be valid and reliable measures, they do have limitations. The self-report approach is subject to measurement errors and misreporting, including deliberate social desirability bias and unintentional bias, such as recall or comprehension error, all of which reduce the precision of the estimate of levels of activity [7-10]. Caution must be taken to select an appropriate physical activity questionnaire according to the purpose of the research and the population under investigation [7].

A major source of bias with self-report questionnaires is the recall period [11]. Typically, self-report measures require participants to remember their physical activities during specific periods of time, such as 3 to 7 days; however, the more distal the recall period, the greater the recall error [12,13]. For example, studies have shown that when using the International Physical Activity Questionnaire (IPAQ), people tended to overreport their physical activity and had difficulty accurately recalling the intensity of the activity done over 1 week [13]. For these reasons, researchers have used diaries or activity logs to record self-reported physical activity on a daily basis to enhance recall [12]. Such approaches require participants to complete paper-based records, which are associated with considerable participant burden and call for sustained cooperation [6].

The ubiquitous use of mobile phones offers a novel approach to measuring physical activity and to reduce participant burden. Mobile phones offer the potential to capture self-report physical activity on a daily basis and offer real-time data collection. Because most people carry a mobile phone most of the time, they have the potential to enhance recall of physical activity by frequent prompting and limiting the time lag between the behavior and data collection. This may reduce information bias and increase compliance; same day or previous day recall has been shown to reduce recall error because error tends to increase with recall duration [12]. Moreover, increased access and availability to mobile phone telecommunications increase the potential of this tool for large-scale data collection in population-based studies [14].

A recent study validated a mobile phone-delivered physical activity questionnaire against doubly labeled water [15]. Twenty-two women reported their physical activity over a 14-day period by answering 2 questions sent daily to their mobile phones. A small mean difference (0.014) with narrow limits of agreement (2 SD 0.30) was found between the mobile phone questionnaire and the reference estimates. In a second study [16], the mobile phone physical activity questionnaire was compared against accelerometry. Both methods showed high within-subject variations; however, the day-to-day variations in energy expenditure within subjects assessed using the mobile phone agreed well with corresponding accelerometer values. The authors concluded that the mobile phone questionnaire was a promising tool for assessing levels of physical activity.

Despite these positive effects, the mobile phone questionnaire has only been examined in healthy Swedish women (aged 20-45 years). Further research is needed to assess the reliability and validity of this questionnaire in males as well as females, with a wider age range. Given the importance of physical activity participation for prevention of chronic diseases and for the secondary prevention of cardiovascular disease (CVD), examination of this approach in a clinical population was warranted. Improving physical activity levels is a key objective following a cardiac event. Not all patients attend structured exercise-based cardiac rehabilitation programs, and they may also exercise in their own time, so it is important that the method used captures habitual activity. The purpose of this study was to determine the convergent validity of a mobile phone physical activity questionnaire against accelerometry in people with CVD. A second aim was to compare how the mobile phone questionnaire performed compared with a commonly used self-recall physical activity questionnaire.

## Methods

### Study Procedures

A 7-day convergent validation study was conducted from January to May, 2012. Participants were recruited from a local exercise-based cardiac rehabilitation clinic in Auckland, New Zealand, and were included if they had documented history of CVD, were currently participating in cardiac rehabilitation, and could safely perform exercise.

The study involved 2 visits. At the first visit, consenting participants completed a demographics questionnaire and underwent a 6-minute walk test (6MWT). Subsequently, participants were temporarily provided with a smartphone and accelerometer. All participants were provided with verbal and written instructions about how to complete the mobile phone physical activity level questionnaire (MobilePAL). Participants responded to 2 physical activity questions initiated by the smartphone application each day for 7 days. The questions were sent to all participants at 7:00 pm each evening. Participants were shown how to properly wear the accelerometer and instructed to wear it for the same 7-day period.

At the end of 1 week, participants completed the second visit, in which they returned the smartphone and accelerometer, and completed a paper copy of the IPAQ.

### Mobile Phone Questionnaire Development

**Figure 1:**
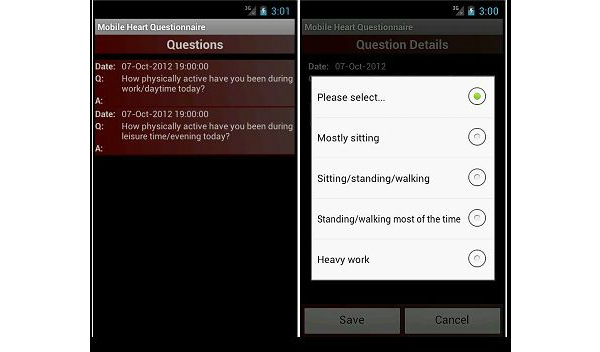
Screenshot of questionnaire and answer categories of the MobilePAL smartphone questionnaire.

For this study, we adapted the original Java-based mobile phone questionnaire [15] for delivery via an Android application (see Figure 1). Smartphone use is increasing, with 44% of people in the United Kingdom [17] and 50% of Americans using smartphones in 2012 [18]. Affordability continues to improve as the cost of smartphones and data plans decrease [19]. Administering the questionnaire by smartphone application offers several advantages over the original Java approach. Participants were not required to have a subscriber identity module (SIM) card or access the Internet to answer the questions, eliminating any costs to the user and reliance on cellular phone networks. Data were saved directly onto the phone in a comma-separated values (CSV) file format, which was then uploaded to a server where it could be safely stored. The application was pretested to ensure the questionnaire functioned and uploaded data correctly.

### Measures

#### Accelerometer

Some consensus exists that accelerometry-based activity monitors provide a useful comparison for evaluating self-report instruments. They can provide detailed information about activity patterns on a minute-by-minute basis and impose only minimal burden on participants [6]. According to Sirard and Pate’s [20] measurement hierarchy, secondary measures such as accelerometers are an acceptable comparison for validating self-report methods. In the present study, accelerometry was used to provide convergent validity against the self-report measures.

Participants wore a dual axial Actigraph GT1M accelerometer (Model AM7164-2.2C Actigraph Ltd, Pensacola, FL, USA), a reliable and valid objective measure of physical activity [21]. The Actigraph is a small, closed device worn at the hip (belt clip or elastic band) that records body movements. Activity counts generated per minute (cpm) were used to determine time spent in light, moderate, and vigorous intensity activity. The following cut-off points were used to determine the intensity of physical activity: sedentary ≤100 cpm, light 101-2020 cpm, moderate 2021-5999 cpm, and vigorous ≥6000 cpm. A valid day consisted of wearing the accelerometer for ≥10 hours of valid time, which was defined as those minutes with <1 hour of consecutive zeros. Average activity counts per minute were calculated for each participant on each valid day, and then averaged over valid days to get the average daily activity count per minute (Acc_CPM). Average daily minutes spent in lifestyle, light, moderate, and vigorous physical activity was also calculated (Acc_PAmin). Activity count data were also used to estimate energy expenditure using the Freedson et al [22] metabolic equivalent (MET) regression equation. METs are multiples of resting metabolic rate during a specific activity, in which 1 MET is equivalent to rest. Daily MET values were calculated by averaging the MET values per minute (converted from raw activity counts per minute using the equation) over each valid day. Average daily METs (Acc_METs) were calculated by summing daily MET values and dividing by the number of days of valid data.

#### MobilePAL Questionnaire

The mobile application presented 2 questions each evening to the user about their physical activity that day, for a total of 7 days. Consistent with previous research [15], for each person and for each day, the answers to the 2 short questions (Table 1) were converted to physical activity level (PAL), which is the ratio between total energy expenditure and resting energy expenditure during 24 hours. The PAL was calculated by combining the PAL values obtained for work/daytime activities reported by Black et al [23] and an additional contribution to PAL from energy expended during leisure/evening activities (Table 1). The latter was calculated from published energy costs expressed as MET values for walking and cycling [24]. PAL data were extracted from the smartphone and imported into Microsoft Excel. Average daily PAL was calculated by summing the daily PAL values and then dividing by 7 days (MobilePAL).

**Table 1:** The two questions administered daily by the smartphone application and their corresponding physical activity level (PAL) value score.

Question	Answer category	PAL score^a^
How physically active have you been during work/daytime today?	Mostly sitting	1.55
	Sitting/standing/walking	1.65
	Standing/walking most of the time	1.85
	Heavy work	2.2
How physically active have you been during leisure time/evening today?	Mostly sitting	+0
	Light/walking (30 min)	+0.06
	Moderate/cycling (>30 min)	+0.15
	Sport/cycling (>60 min)	+0.29

^a^Daily PAL was calculated by combining the PAL score from question 1 (work/daytime physical activity) and question 2 (leisure time/evening physical activity).

#### International Physical Activity Questionnaire (IPAQ)

The IPAQ is a reliable and validated 7-day recall measure, which provides a comprehensive evaluation of daily physical activities, and assesses time spent walking and doing light, moderate, and vigorous intensity activities across various domains [24]. Computation of the total scores required summation of the duration (in minutes) and frequency (days) for all the types of activities in all domains. Domain specific scores were calculated by summing the scores for walking, moderate, and vigorous intensity activities within the specific domain. Activity-specific scores were calculated by summing the scores for the specific type of activity across domains.

Two variables were derived from the IPAQ data: (1) average daily active minutes (IPAQ_PAmin), which was calculated by summing total time spent walking, in moderate, and in vigorous intensity physical activity, and then dividing by 7 days; (2) average daily physical activity level (IPAQ_met), which was calculated using total physical activity (MET-minutes per week) divided by 7 days. MET-minutes per week were derived as duration×frequency per week×MET intensity assigned to each category of activity [25].

#### Six-Minute Walk Test (6MWT)

The 6MWT is a test of physical capacity commonly used in the assessment of cardiac rehabilitation patients and was used to assess functional capacity of participants. Each participant completed the test once during their first study visit. The 6MWTs were administered by a research assistant using a standard protocol [26].

### Analysis

Statistical analyses were performed using SAS version 9.2 (SAS Institute, Inc, Cary, NC, USA) and R version 2.15.0 (R Foundations for Statistical Computing, Vienna, Austria). All statistical tests were 2-tailed at a 5% significance level. Participants’ characteristics and physical activity measurements were first summarized using descriptive statistics. Both Pearson product moment correlation (*r*) and Spearman rank order correlation were used to assess the strength of correlation between 2 measures with associated *P* values. Regression analyses were carried out to investigate the relationships between the MobilePAL questionnaire, the accelerometer, and self-report physical activity measured by the IPAQ. For accelerometer data, Acc_CPM was compared with MobilePAL because this captures all activities performed by the person when wearing the device, including incidental and lifestyle activities. As stated, we estimated average daily METs (Acc_METs), which reflects activity-related energy expenditure. In the present study, we did not collect body mass data; therefore, we were unable to estimate basal metabolic rate and could not truly estimate PAL. The Acc_METs was considered an appropriate proxy measure for comparison with MobilePAL. Energy expenditure obtained from the IPAQ (IPAQ_met) was chosen as a comparator because it includes a similar measurement unit as MobilePAL. Potential confounding effects of age and functional capacity (6MWT) were examined in all models. Repeated measures analysis was also conducted to evaluate the change in PAL over the 7-day period.

A Shapiro-Wilk test of normality was conducted with the relatively small sample size (N=30). When necessary, log transformation of the outcome variable was considered. Because the results with and without the log transformation were similar in all analyses, the original (nontransformed) data are presented.

## Results

As shown in Table 2, most participants were New Zealand European (29/30, 97%), men (26/30, 87%), aged between 49 to 85 years (mean 65.6, SD 8.8). Thirty-six potential participants were approached to take part. Of these, 32 expressed interest and 30 completed the study (30/36, 83%). More than half were working full or part time (19/30, 63%) and 11/30 (37%) were retired. Thirteen participants (43%) had never smoked and 17/30 (57%) identified as previous smokers. Twenty-one participants (70%) consumed at least 1 alcoholic drink per week. Distance walked during the 6MWT ranged from 372 to 742 meters (mean 570.8, SD 96.3).

Descriptive summaries of all physical activity measurements obtained by the 3 different instruments are presented in Table 3. All participants provided at least 4 days of valid accelerometer data. Average daily active minutes measured by IPAQ (IPAQ_PAmin) were on average lower than the average daily valid minutes recorded by accelerometer (Acc_PAmin), which is not surprising because the IPAQ does not capture incidental movement.

**Table 2:** Participant characteristics (N=30).

Variables		Participants n (%)
**Gender**		
	Male	26 (87)
	Female	4 (13)
**Ethnicity**		
	New Zealand European	29 (97)
	Māori (indigenous)	1 (3)
**Medical status^a^**		
	High blood pressure	23 (77)
	High cholesterol	26 (87)
	Diabetes	5 (17)
	Atrial fibrillation	10 (33)
	Heart attack	14 (47)
	Angina	3 (10)
	Other forms of heart disease	12 (40)

^a^Some participants reported having more than 1 medical condition.

**Table 3:** Summary of average daily physical activity obtained by the 3 different instruments, the smartphone, the accelerometer, and the International Physical Activity Questionnaire (IPAQ).

Instrument and measurement		Mean (SD)	Range
**Smartphone^a^**			
	MobilePAL	1.77 (0.1)	1.6-2.1
**Accelerometer^b^**			
	Acc_CPM	313 (140)	108-702
	Acc_METs	1.69 (0.1)	1.5-2.0
	Acc_PAmin	302 (74)	188-528
**IPAQ^c^**			
	IPAQ_met	531 (468)	47-1840
	IPAQ_PAmin	149 (131)	14-504

^a^MobilePAL=daily physical activity level (PAL) measured by smartphone questionnaire.

^b^Acc_CPM=daily activity counts per minute measured by accelerometer; Acc_METs=average daily metabolic equivalent derived from accelerometer counts per minute; Acc_PAmin=daily minutes of lifestyle, light, moderate, or vigorous physical activity measured by accelerometer.

^c^IPAQ_met=MET-minutes per day measured by IPAQ; IPAQ_PAmin=daily minutes of walking, in moderate intensity, and in vigorous intensity physical activity measured by IPAQ.

The Pearson’s correlation coefficients are presented in Table 4. Associations between the MobilePAL questionnaire and accelerometer daily activity counts (Acc_CPM), activity-derived METs (Acc_METs), and IPAQ-derived energy expenditure (IPAQ_met) were similar in magnitude. The conclusions were consistent using Spearman rank correlation and, therefore, are not reported here. Graphs illustrating the degree of spread in the data are shown in Multimedia Appendix 1. Functional capacity measured by the 6MWT was not associated with any variables except age (*r*=–.43; *P*=.02). Age was not associated with the self-report measures, but showed moderate correlations with Acc_CPM (*r*=–.53; *P*=.003) and Acc_PAmin (*r*=–.46; *P*=.01).

**Table 4:** Correlations between 3 different instruments, the smartphone, the accelerometer, and the International Physical Activity Questionnaire (IPAQ).

Instrument and measurement		Correlations, *r* ^a^					
		MobilePAL	Acc_CPM	Acc_METs	Acc_PAmin	IPAQ_met	IPAQ_PAmin
**Smartphone^b^**							
	MobilePAL	1.00					
**Accelerometer^c^**							
	Acc_CPM	*.45*	1.00				
	Acc_METs	*.45*	*1.00*	1.00			
	Acc_PAmin	*.39*	*.81*	*.81*	1.00		
**IPAQ^d^**							
	IPAQ_met	*.49*	*.62*	*.62*	*.40*	1.00	
	IPAQ_PAmin	*.48*	*.61*	*.61*	*.41*	*.99*	1.00

^a^Statistically significant correlations (*P*<.05) are indicated in italics.

^b^MobilePAL=daily physical activity level (PAL) measured by smartphone questionnaire.

^c^Acc_CPM=daily activity counts per minute measured by accelerometer; Acc_METs= Average daily metabolic equivalent derived from accelerometer counts per minute; Acc_PAmin=Daily minutes of lifestyle, light, moderate, or vigorous physical activity measured by accelerometer.

^d^IPAQ_met=MET- minutes per day measured by IPAQ; IPAQ_PAmin=daily minutes of walking, in moderate intensity, and in vigorous intensity physical activity measured by IPAQ.

**Figure 2:**
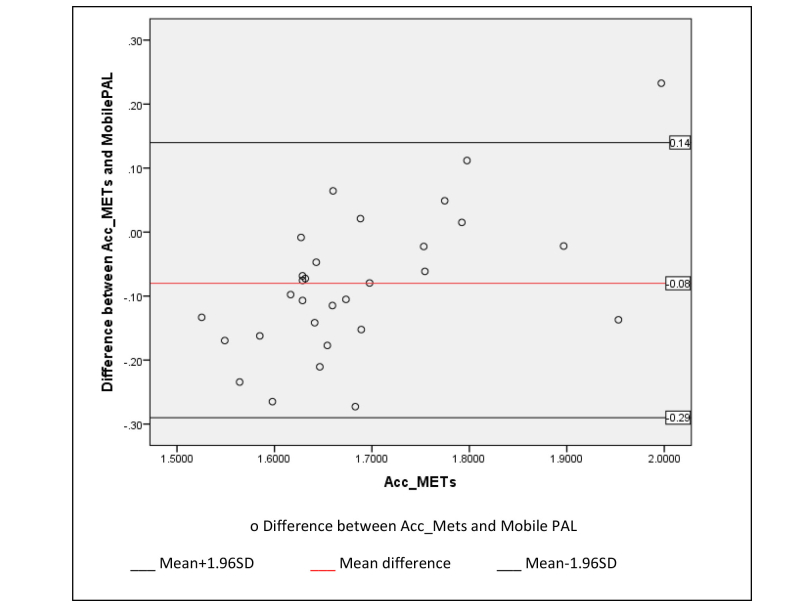
Bland-Altman plot comparison of energy expenditure obtained using accelerometry (Acc_METs) and the mobile phone questionnaire (MobilePAL).

To determine the level of agreement between accelerometer-derived energy expenditure (ACC_METs) and Mobile PAL we conducted a Bland-Altman plot (see Figure 2). Overall, there was good agreement between the methods, with a mean bias of +0.08 METs.

Linear regression analyses were used to further investigate the relationships between the variables of interest. Because 6MWT was not associated with any of these variables, only age was adjusted in the regression analysis.

We first examined the association between MobilePAL and the reference standard of accelerometry (Acc_CPM). Both MobilePAL (beta=.42; *P*=.008) and age (beta=–.48; *P*=.002) were significant predictors of Acc_CPM (adjusted *R^2^*=.40, *F*_2,27_=10.58; *P*<.01). Because Acc_METs were derived from accelerometer cpm, a separate regression equation was not conducted. Similar findings were found when IPAQ-derived energy expenditure was considered in the model. Both IPAQ_met (beta=.51; *P*=.001) and age (beta=–.36; *P*=.016) made unique contributions to the predicted model (adjusted *R^2^*=.47, *F*_2,27_=13.58; *P*<.001). We also found that IPAQ_met (beta=.51; *P*=.007) was strongly associated with MobilePAL (adjusted *R^2^*=0.19, *F*_2,27_=4.32; *P*<.05), but not age (beta=.07; *P*=.682).

Repeated measures analysis on MobilePAL over 7 days revealed little within-day variability. No differences in MobilePAL between any 2 days were observed after Tukey-Kramer adjustment, except for days 6 and 7 (adjusted *P*=.04).

## Discussion

We found relatively good associations between the mobile phone-derived activity-related energy expenditure (MobilePAL) and the accelerometer-derived total daily activity counts (Acc_CPM), which was similar to that observed with a commonly used self-report questionnaire, the IPAQ, and slightly better than that demonstrated in the 12-country validation of the IPAQ [25]. The similar magnitude of correlations for IPAQ and MobilePAL with accelerometry data indicates the mobile phone questionnaire is as good as traditional paper-based questionnaires. There is potential that the mobile phone questionnaire may have enhanced recall on the IPAQ, as participants were answering questions each day about their physical activities. This constant recall may have led to stronger associations. Good agreement existed between mobile phone and accelerometer derived activity-related energy expenditure (MobilePAL and Acc_METs) with only slight overestimation of the mobile phone questionnaire compared to the reference standard of accelerometry. Taken together, these findings support the use of the mobile phone questionnaire to assess physical activity levels in people with CVD.

Our study sample included attendees of a cardiac rehabilitation program; however, many people with CVD are encouraged to exercise but do not attend cardiac rehabilitation programs [27-30]. Moreover, adherence at cardiac rehabilitation is low and many people exercise on their own time in their community. This mobile phone questionnaire offers a viable approach to monitoring activity levels of people with CVD, irrespective of attendance at cardiac rehabilitation. Such data are needed to truly understand the benefits of physical activity for secondary prevention of CVD.

In comparison to other cardiac rehabilitation populations, our sample had a similar mean age [31]. In the present study, age was a significant predictor in the regression analyses; the older participants in our sample were less active than the younger participants, as revealed by the inverse relationship between accelerometer counts per minute and age. Moreover, participants in our study had better physical function with a mean 6MWT of 570 meters, which is greater than that observed in other postcardiac rehabilitation populations (377-555 m) [31]. This may be due to a higher exercise dose as our sample participated in a supervised exercise program 3 times per week.

The repeated measures analyses showed little within-day variability for the mobile phone questionnaire. For 28 participants, day 6 fell on a Tuesday (n=23) or a Thursday (n=5). The study participants belonged to a cardiac rehabilitation exercise clinic that ran on Mondays, Wednesdays, and Fridays, so perhaps the lower PAL score on day 6 was a reflection of a rest day for some participants.

This study builds on previous validation research by Bexelius and colleagues [15] by testing the convergent validity of the mobile phone questionnaire in people with CVD. Previously, the questionnaire has demonstrated validity against doubly labeled water and accelerometry in Swedish women. Our findings extend the generalizability of this questionnaire; taken together, these findings suggest that this mobile phone questionnaire is a reliable and valid self-report measure of physical activity.

Recently, another mobile phone-based physical activity questionnaire has been developed. Sternfeld and colleagues [32] evaluated an activity diary as an application program to be administered using a mobile phone. Participants were asked to record their physical activities on their phone 3 times a day. Participants could choose activities across 15 different domains, and responses were associated with MET values derived from the Compendium of Physical Activities [24]. Compared to accelerometry, intraclass correlation coefficients ranged from 0.55 for light physical activity to 0.63 for vigorous activity, whereas correlations were of moderate magnitude and slightly higher than observed in the present study. As with our study, there was good user acceptability. Collectively, these studies highlight the utility of mobile phones for self-reporting physical activity.

Despite these findings, it is important that researchers do not necessarily develop a completely new series of mobile phone applications for self-reporting physical activity, but rather build on previous research by refining existing platforms. This would avoid the unnecessary proliferation of questionnaires observed in the field of physical activity research. To illustrate, there are currently more than 100 self-report measures of physical activity in use, with varying degrees of reliability and validity. A recent review by Helmerhorst and colleagues [7] identified 34 physical activity questionnaires developed between 1997 and 2011, and found that these were no more reliable or valid compared to existing measures. We acknowledge that the field of physical activity measurement and the resulting techniques and analytic approaches has progressed considerably as a function of the development of these questionnaires. However, we suggest that as researchers, it is in our best interest not to continue this trend of proliferation with mobile phone questionnaires. A more fruitful approach might be to build on existing measurement expertise and work to refine or develop a universal mobile phone questionnaire for population-level use, as has been done with the IPAQ or Global Physical Activity Questionnaire.

An important finding from this study is that our sample of middle-aged to older adults were able to use the smartphone application. The digital divide, whereby some groups (including older-aged people) may use technology less than others, has been considered a barrier for researchers using smartphone applications [19]. However, there is now abundant evidence that mobile phones offer unprecedented opportunities to improve reach into traditionally underserved population groups [33,34]. Indeed, the telecommunications industry has documented a trend toward a digital divide in reverse, whereby low income and ethnic minority groups use the technology more than others [14]. It is estimated that 80% to 90% of the UK population will have a smartphone within 10 years [19]. Given this increasing use and availability of smartphone technology, combined with real-time data collection that is easy to use, makes this a promising way to obtain physical activity data and retain participant compliance. Participants are required to answer only 2 questions, thereby reducing the burden typically associated with other commonly used instruments. It takes less than 30 seconds to answer the MobilePAL questionnaire each day, whereas the IPAQ took our participants between 10 to 15 minutes to complete. Previous research has highlighted the need to make mobile phone applications easy to use (ie, 1 click from main page) [35]. This was an important consideration in our application development; hence, participants required a single click from the main page to access the application, 1 click to select answers to the questions, and 1 click to save responses. Most participants had never used a smartphone prior to this study, and anecdotal responses indicated that they generally found the application easy to use. Collectively, these features make mobile phone questionnaires, such as the one presented here, suitable for population-level surveillance.

This study is not without limitations. First our findings are based on a small (N=30) and relatively homogeneous sample of New Zealand European men, which impacts on the variability and generalizability of the data. However, the mobile phone questionnaire has been validated in other populations using doubly labeled water and indirect calorimetry [15]. Welk [6] describes validity as an ongoing and community process because validation cannot be determined by one study alone. Future research is needed to validate this questionnaire in other subgroups and countries.

In conclusion, a mobile phone–delivered questionnaire is a relatively reliable and valid measure of physical activity, and is as good as existing self-report measures. Given their ubiquitous use, mobile phones may be an effective method of physical activity surveillance data collection.

## Multimedia Appendix 1

Degree of spread between variables.

## Abbreviations

6MWT: 6-minute walk test
Acc_CPM: daily activity counts per minute measured by accelerometer
Acc_METs: average daily metabolic equivalent derived from accelerometer counts per minute
Acc_PAmin: daily minutes of lifestyle, light, moderate, or vigorous physical activity measured by accelerometer
CSV: comma-separated values
CVD: cardiovascular disease
IPAQ: International Physical Activity Questionnaire
IPAQ_met: MET-minutes per day measured by IPAQ
IPAQ_PAmin: daily minutes of walking, in moderate intensity, and in vigorous intensity physical activity measured by IPAQ
MET: metabolic equivalent
MobilePAL: daily physical activity level (PAL) measured by smartphone questionnaire
PAL: physical activity level
SIM: subscriber identity module
