# Supplementary figures and images for: Measuring Physical Activity in a Cardiac Rehabilitation Population Using a Smartphone-Based Questionnaire
Source: J Med Internet Res. 2013 Mar 22;15(3):e61. doi: 10.2196/jmir.2419 (PMC3636157; doi:10.2196/jmir.2419)

## Appendix 1. Degree of spread between variables

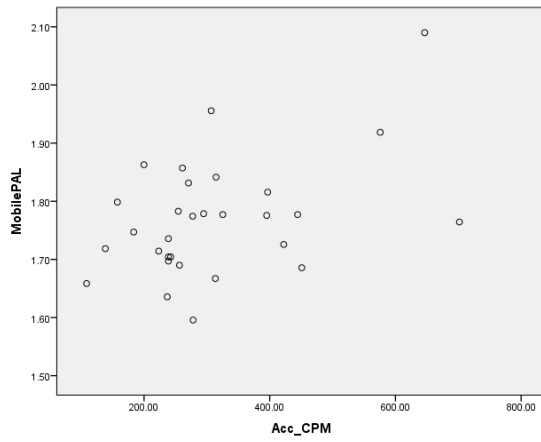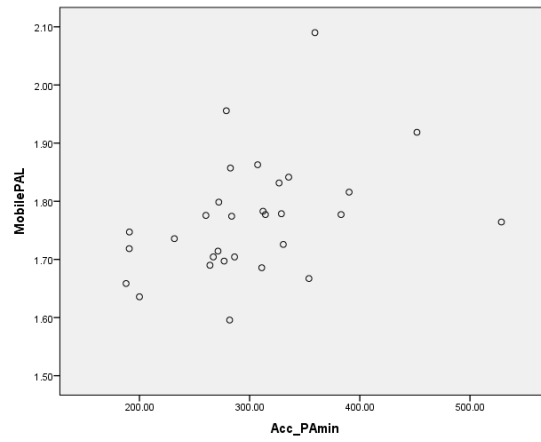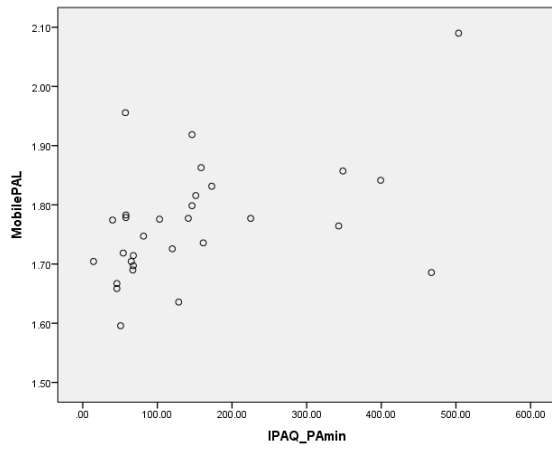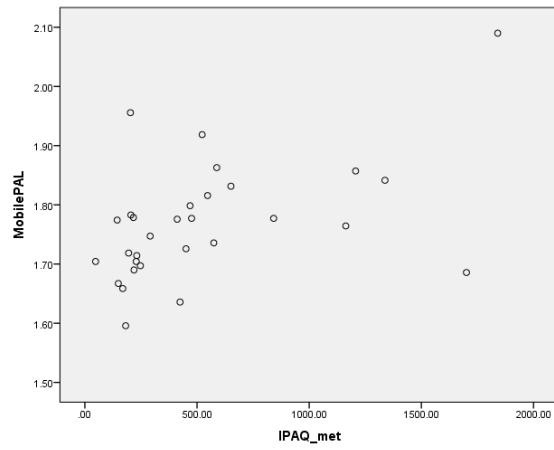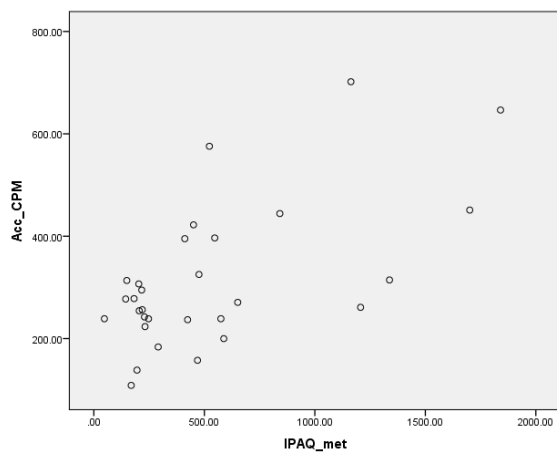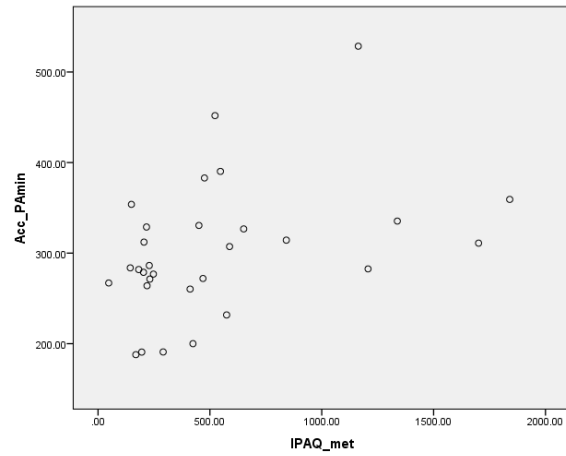

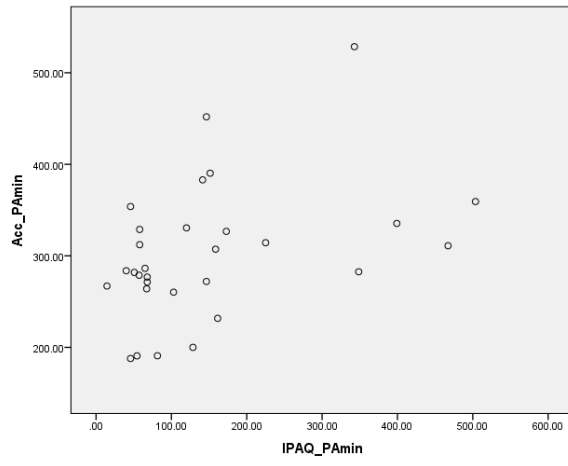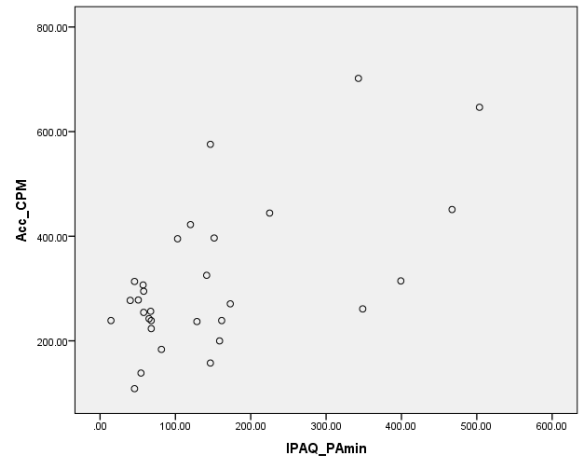

Supplement: Supplementary file 1 [file jmir_v15i3e61_app1.pdf]
